# Mitotic Acetylation of Microtubules Promotes Centrosomal *PLK1* Recruitment and Is Required to Maintain Bipolar Spindle Homeostasis

**DOI:** 10.3390/cells10081859

**Published:** 2021-07-22

**Authors:** Sylvia Fenosoa Rasamizafy, Claude Delsert, Gabriel Rabeharivelo, Julien Cau, Nathalie Morin, Juliette van Dijk

**Affiliations:** 1 Université de Montpellier, 34293 Montpellier, France; sylvia.rasamizafy@crbm.cnrs.fr (S.F.R.); claude.delsert@crbm.cnrs.fr (C.D.); gabriel.rabeharivelo@crbm.cnrs.fr (G.R.); julien.cau@igh.cnrs.fr (J.C.); 2 Centre National de la Recherche Scientifique (CNRS) UMR5237, 1919 Route de Mende, 34293 Montpellier, France; 3 Institut Français de Recherche pour l’Exploitation de la mer, L3AS, 34250 Palavas-les-Flots, France; 4 IGH, CNRS UMR 9002, 141, rue de la Cardonille, 34396 Montpellier, France; 5 Montpellier Rio Imaging, 34293 Montpellier, France

**Keywords:** microtubules, acetylation, acetyltransferase *ATAT1*, mitosis, spindle, centrosome, kinetochore, *PLK1* kinase

## Abstract

Tubulin post-translational modifications regulate microtubule properties and functions. Mitotic spindle microtubules are highly modified. While tubulin detyrosination promotes proper mitotic progression by recruiting specific microtubule-associated proteins motors, tubulin acetylation that occurs on specific microtubule subsets during mitosis is less well understood. Here, we show that siRNA-mediated depletion of the tubulin acetyltransferase *ATAT1* in epithelial cells leads to a prolonged prometaphase arrest and the formation of monopolar spindles. This results from collapse of bipolar spindles, as previously described in cells deficient for the mitotic kinase *PLK1*. *ATAT1*-depleted mitotic cells have defective recruitment of *PLK1* to centrosomes, defects in centrosome maturation and thus microtubule nucleation, as well as labile microtubule-kinetochore attachments. Spindle bipolarity could be restored, in the absence of *ATAT1*, by stabilizing microtubule plus-ends or by increasing *PLK1* activity at centrosomes, demonstrating that the phenotype is not just a consequence of lack of K-fiber stability. We propose that microtubule acetylation of K-fibers is required for a recently evidenced cross talk between centrosomes and kinetochores.

## 1. Introduction

Microtubules (MTs) are long hollow tubes made of 13 protofilaments, formed by the polymerization of αβ-tubulin heterodimers [1]. In cells, elongation and shrinkage of MTs results from polymerization/depolymerization of tubulin subunits. Controlled and differential regulation of MT dynamics results in the arrangement of strikingly different MT networks, allowing specific cell functions. Examples are the long bundled MTs which regulate the elongation of axons in neurons, while, in mitosis, chromatid alignment and their subsequent faithful separation depends upon the organization of MTs into a mitotic spindle.

MT behavior and specialization requires binding of microtubule-associated-proteins (MAPs) and molecular motors. It is now generally accepted that specific tubulin isotypes and post-translational modifications (PTMs) mark MTs for spatial and temporal recruitment of specific MAPs and motors, exemplifying the “tubulin code” [2].

Accordingly, it was shown that the MT severing activity of the spastin ATPase, altered in autosomal dominant spastic paraplegia [3], is strictly controlled by MT polyglutamylation [4,5]. The tubulin code has been further supported by the discovery that, in mitosis, selective MAP and motors are recruited by subsets of either tyrosinated or detyrosinated MTs to promote spindle orientation and proper chromatid alignment to the metaphase plate [6,7,8].

The unstructured C-terminal tail of MTs is the target of most PTMs but this is not the case for MT acetylation that occurs on lysine 40 (α-tubulin K40) located in the lumen of MTs [9]. MT acetylation was long considered as a marker of MT stability, but recent studies now show that MT acetylation increases MT flexibility and resistance to mechanical breakage by weakening the interactions between protofilaments, thus increasing the microtubule lifespan [10,11].

Although the MT acetyltransferase *ATAT1* is not essential for embryo survival in mice [12,13,14], MT acetylation regulates intracellular transport [15], lysosome trafficking [16], and focal adhesion dynamics [17,18] and is involved in multiple processes such as dendrite and axon morphogenesis in neurons [19], cell–cell contact inhibition [13], cell invasion and migration [20,21]. This apparent discrepancy between essential functions of *ATAT1* at the cellular level and viable knockout embryos may be reconciled by mounting evidence that *ATAT1* may be crucial under altered physiological conditions revealed in cell culture, yet its loss can be compensated for by other mechanisms in the natural tissue environment [13,22].

The MT acetylation levels vary from one cell type to the other, allowing specific cellular functions, but these differences are lost during mitosis when spindles are assembled [23]. This suggests the need for a tight regulation of MT acetylation in mitosis. During spindle evolution, different subsets of MTs are acetylated [24,25]. In prophase and prometaphase, acetylation is mainly present on the centrosomes. At metaphase and during anaphase, acetylation appears on the spindle MTs but is excluded from astral MTs. The midbody gets acetylated only when microtubule compaction has occurred. Although mitotic microtubule acetylation is highly dynamic, little is known about its functions. Interference with several regulators of the MT deacetylase HDAC6, such as Kindlin, RITA, or ESWR1, induces mitotic defects correlated with deregulated MT acetylation [22,25,26,27]. However, function of *ATAT1*-mediated acetylation of mitotic spindle microtubules remains unknown. *ATAT1* knockout mice are viable but low levels of acetylated tubulin remain on the mitotic spindles of *ATAT1*^−/−^ murine embryonic fibroblasts [13], indicating that another acetyltransferase may overcome the lack of *ATAT1*.

In this study, we investigated *ATAT1* functions in mitosis in an epithelial cell model. Loss of *ATAT1* induced severe mitotic defects that are characterized by a prolonged prometaphase-like arrest and formation of monopolar MT spindles. The G2/M activity of kinesin-5 was not downregulated in *ATAT1* depleted cells. Instead, monopolarization occurs by spindle collapse after initial centrosome separation. We observed that spindles induced in either *ATAT1*-depleted or *PLK1*-inhibited cells present striking similarities. *PLK1* is a major mitotic kinase that regulates MT nucleation and dynamics throughout the spindle [28,29]. We found that, in *ATAT1*-depleted mitotic cells, *PLK1* recruitment to the centrosomes was strongly reduced. We could rescue the monopolar spindle phenotype of *ATAT1*-depleted cells by stabilizing spindle MTs by different means or by codepleting *ATAT1* with MYPT1, the regulatory subunit of myosin phosphatase [30] which regulates mitotic centrosomal *PLK1* activity [31]. Thus, we provide evidence that *ATAT1*-dependent MT acetylation regulates centrosomal *PLK1*.

## 2. Materials and Methods

### 2.1. Antibodies and Reagents

Rabbit antibodies against tubulin C102 were a gift of Dr. M. Andreu and *HEC1* antibodies were a gift of Dr. PT. Stuckenberg (University of Virginia, Charlottesville, VA, USA). Polyclonal antibodies against *TPX2* and PolyE-Tubulin were home-made [32,33]. All other antibodies are commercially available: Acetyl-tubulin 6-11B-1 (Santa Cruz, Santa Cruz, CA, USA), α-tubulin DM1A (Sigma, Burlington, MA, USA), Vinculin (Sigma), γ-tubulin *GTU88* (Sigma), Kinesin 5 (Abcam, Cambridge, UK), pericentrin (Abcam), NEDD1 (Abcam), PRC1 (Santa Cruz), HURP (Proteintech, Chicago, IL, USA), CREST (Europa Bioproducts, Cambridge UK), *PLK1* clone 35-206 (Millipore, Burlington, MA, USA), KIF2A (Novus, Littleton, CO, USA), NuMA (Abcam), *BUBR1* (Cell Signaling, Danvers, MA, USA), *MCAK* (Millipore), *MYPT1* (Proteintech).

Most chemicals, including Nocodazole, STLC, and MG132, were obtained from Sigma. BI2356 was purchased from MedChemExpress.

### 2.2. Cell Culture and Generation of Cell Lines

Pig kidney epithelial LLCPK1 cells were maintained at 37 °C in a 5% CO_2_ atmosphere in Dulbecco’s modified medium (DMEM) supplemented with 10% fetal bovine serum (GE Healthcare, Chicago, IL, USA), 100 U/mL penicillin, and 100 U/mL Streptomycin.

GFP-α-Tubulin LLCPK1 stable cell line [34] was a gift from B. Delaval (CNRS, Montpellier, France).

LLCPK1 cells expressing Eos3.2-tubulin were generated using the mEos3.2-Tubulin-C-18 plasmid kindly given by Michael Davidson ([35], Addgene # 57484). Plasmid was transfected in LLCPK1 cell using lipofectamin plus according to manufacturer instructions (Thermofisher Scientific, Illkirch, France) and stable clones were selected using G418 at 1.5 mg/mL.

For generation of LLCPK1 cells expressing doxycycline-inducible *ATAT1* (wt or Dead)-GFP, mus musculus *ATAT1*-GFP was cloned into the Sfi cleavage site of the pSBtet-Pur vector ([36], Addgene #60507). The “Dead” version was generated using the QuickChange Site-directed mutagenesis kit (Agilent Technologies, Santa Clara, CA, USA) in order to introduce the G469A mutation (D157N) that kills the acetyltransferase activity of *ATAT1* [37]. These constructs are not sensitive to the siRNA against sus scrofa ATAT-1. Plasmids were transfected in LLCPK1 cells using lipofectamin plus according to manufacturer instructions (Thermo Fisher Scientific) and stable clones were selected with puromycin. Expression of transgenes was initiated by addition of 1 µg/mL doxycycline.

### 2.3. siRNAs, Cell Transfection, and Drug Treatments

To deplete luciferase (control) and *ATAT1* from LLCPK1 cells, we respectively used Eurogentec synthesized siRNA 5′-CGUACGCGGAAUACUUCGA(dT)(dT)3′ which is not homologous to anything in vertebrate transcriptomes and siRNA 5′-CCACACCAGCUGGCUAUUGA(dT)(dT)3′ targeting nt 451–470 from ATG of sus scrofa *ATAT1*. SiRNAs (50 nM) were transfected for 48 h using RNAiMAX (Invitrogen, Waltham, MA, USA). In some cases, cells were enriched in mitotic cells by overnight treatment with 2 µM STLC 24 h after siRNA transfection followed by a release for 2 h in 10 µM MG132. The *PLK1* inhibitor BI2356 was used at 100 nM for 2 h. For nocodazole treatments, two different concentrations were used. To induce complete MT depolymerization (Figure 3c), cells were treated with 1 µg/mL for 1 h. To stabilize MTs (Figure 7a), 10 or 20 ng/mL were used. For cold treatment, cells were rinsed and incubated in cold medium for indicated times at 4 °C.

### 2.4. Immunofluorescence

Cells were fixed with 4% paraformaldehyde in PEM buffer (0.1 mM PIPES, pH 6.9; 1 mM EGTA; 0.5 mM MgCl_2_) containing 0.2% Triton X-100 for 10 min at 37 °C or in MeOH for 10 min at −20 °C, blocked with 1% BSA and stained with appropriate antibodies diluted in PBS containing 5% fetal calf serum for 1 h, then washed 5 times in PBS and incubated with either Alexa-350, -488, -555 or -640 conjugated secondary antibodies directed against different species (Life Science, Saint Petersburg, FL, USA), then washed 5 times in PBS. Cells were mounted in ProLong Gold Antifade Mountant (Invitrogen).

### 2.5. Cell Imaging

Fixed cell imaging was performed with a Zeiss LSM880 Airyscan confocal microscope equipped with a ×63 Apo 1.4NA oil objective. Images were acquired using low laser power. Spatial resolution was improved by the use of the airyscan module. Most images are Maximal Intensity Projections (MIP) of 3D Z stacks. Sometimes single planes are shown, as indicated. Same laser power was used in the different siRNA conditions to be able to compare intensities of antibody stainings.

For live imaging of GFP-tubulin LLCPK1 cells, Z stack images were recorded on a dragonfly ANDOR spinning disk confocal microscope equipped with a ×60 Apo lambda 1.4NA DT oil objective and a EMCCD iXon888 Life Andor camera.

For flux measurements, Eos3.2-tubulin-expressing LLCPK1 cells were imaged on a LSM780 confocal microscope with an opened pinhole (3.5 AU). A defined region was photoactivated at 405 nm (one scan, dwell time 2.5 µs, 40 iterations, 100% laser) and images (channel S1 472–526 and channelS2 561–565) were acquired every 10 s.

### 2.6. Image Quantification and Statistical Analysis

The mitotic index was determined by the “analyze particle” function of ImageJ software using DAPI staining. For quantification of the time spent between NEBD and anaphase onset, live GFP-tubulin-expressing LLCPK1 cells were imaged. Timing of NEBD was defined by the loss of nucleus contrast and appearance of MT staining filling the nuclear area and timing of anaphase onset was determined by elongation of the spindle.

For phenotype analyses, the number of monopolar, bipolar, and eventually multipolar spindles were manually counted. Distances between centrosomes were measured on 3D stacks using Imaris software. For si*ATAT1*, only cells with focused centrosomes were analyzed.

For quantification of the total fluorescence intensities of γ-tubulin, pericentrin, NEDD1, *TPX2*, and tubulin, we used the ImageJ software. Images were first segmented using an intensity threshold and unresolved clusters of cells were discarded. Centrosome’s regions were defined manually or with the « analyze particle » function of ImageJ. Total fluorescence intensity was calculated by multiplying the area by the mean intensities.

Quantification of total *HEC1* and *PLK1* intensities at centrosomes and at every kinetochore was performed on 3D stack images defining volumes for centrosomes and spots for kinetochores using Imaris software. Automatization was done using the batch convert function.

Flux speed was calculated by measuring the shortening of the distance from the spindle pole to the middle of the photoactivated tubulin signal as a function of time.

Quantification of NuMA dots occurring on K-fibers, away from the spindle pole region, was performed using the 3D stack images. Individual K-fibers were manually tracked and NuMA dots were counted. Occurrence of dots per µm was then calculated.

For KT-MT attachment, tubulin and CREST stainings were used. 3D images of mitotic spindles were analyzed using Imaris software. KTs were counted using the « Add Spots » function and assessed for binding to K-fibers.

All quantifications were plotted in Graphpad Prism software. The number of cells analyzed and the number of independent experiments (*n* ≥ 3) are indicated in figure legends. Statistical calculations were performed using unpaired t-tests. *p* < 0.0001 is considered highly significant and noted in the graphs with ****. P Values between 0.0001 and 0.001 are annotated with ***; between 0.001 and 0.01 with ** and between 0.01 and 0.05 with *. Not significant differences (*p* ≥ 0.05) are indicated by ns. Error bars are SEM of mean.

## 3. Results

### 3.1. Loss of ATAT1 Induces Mitotic Defects in LLCPK1 Cells

**Figure 1:**
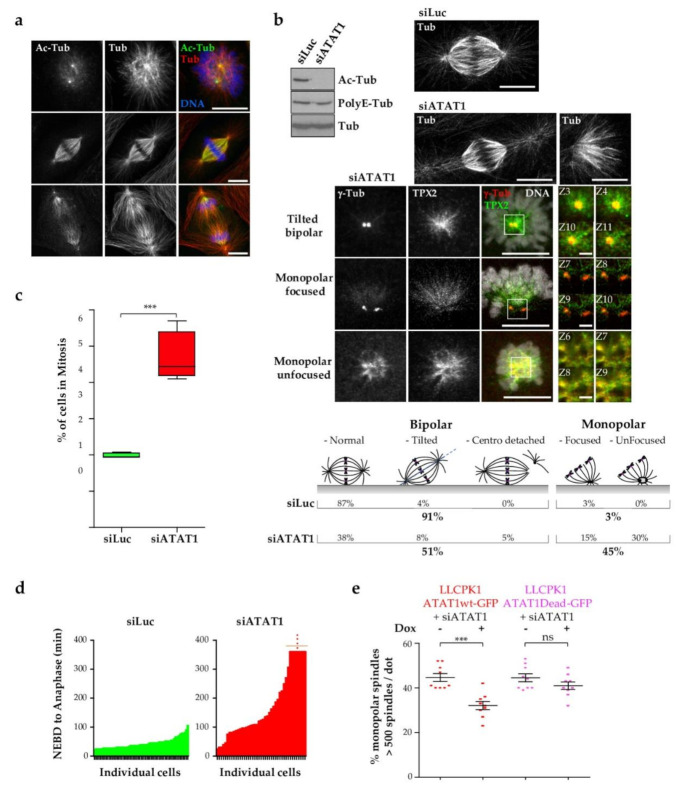
*ATAT1* depletion induces mitotic defects. (**a**) Maximum intensity projection (MIP) images of mitotic LLCPK1 cells stained for acetyl-tubulin (Ac-Tub), tubulin (Tub) and DAPI. Different subsets of MTs are stained for Ac-Tub in prometaphase, metaphase, and anaphase. Scale bars are 10 µm. (**b**–**d**) LLCPK1 cells transfected for 48 h with siRNAs directed against luciferase (Luc) and *ATAT1*. (**b**) Cell lysates were analyzed by western blot using Ac-Tub and PolyE-tubulin antibodies. Total tubulin antibody was used as loading control. Representative MIP images of tubulin-stained mitotic Luc- and *ATAT1*-depleted cells as well as *ATAT1*-depleted cells stained for γ-tubulin and *TPX2* are shown. Scale bars are 10 µm. Insets are single planes focusing on the centrosome region. Z section, as indicated shows that centrosomes may be in same or different planes. Z step is 0.18 µm. Scale bar is 2 µm. Cartoon illustrates the quantification of spindle phenotypes that were manually analyzed in siLuc and si*ATAT1* cells. 100 to 300 spindles were analyzed per condition and per experiment (*n* = 3). (**c**) Mitotic index of siLuc and si*ATAT1* cells. 1000 to 5000 cells were counted per condition and per experiment (*n* = 4). (**d**) Quantification of the time spent by siLuc and si*ATAT1* cells between nuclear envelop breakdown (NEBD) and initiation of anaphase onset. Live imaging of GFP-Tubulin-expressing LLCPK1 cells transfected with Luc or *ATAT1*-siRNAs was started 48 h after transfection. Images were acquired every 3 min and about 15 mitoses were analyzed per condition and per experiment (*n* = 3). (**e**) Inducible GFP-wild type (wt) and Dead *ATAT1*-LLCPK1 cells were transfected for 48 h with Luc and *ATAT1* siRNAs. Doxycycline (Dox) was or not added 24 h post transfection to induce expression of GFP-wt/Dead-*ATAT1* transgenes. Over 500 fixed mitotic cells stained for tubulin and γ-tubulin were manually analyzed, per condition and per experiment, for their spindle phenotype (*n* = 9). *p* Values between 0.0001 and 0.001 are annotated with ***.

As previously reported, several MT subsets are highly acetylated during mitotic spindle evolution (Figure 1a, [38]). To study the role of MT acetylation in mitotic progression, we depleted the MT acetyltransferase *ATAT1* by RNA interference in LLCPK1 epithelial cells. Figure 1b shows that *ATAT1*-depleted cells (si*ATAT1* cells) have undetectable levels of acetyl-tubulin while MT polyglutamylation and total tubulin levels are not affected. Mitotic failure was observed in about 50% of the *ATAT1*-depleted cells, the most prominent phenotype being the formation of monopolar spindles as revealed by tubulin staining. In addition, spindles with detached centrosomes were seen (Figure 1b). To better observe centrosomes of *ATAT1*-depleted MT spindles, we co-stained si*ATAT1* cells for the centrosomal component γ-tubulin and the microtubule-associated protein *TPX2* which is crucial for spindle formation. γ-Tubulin staining of *ATAT1*-depleted spindles can be resolved as two dots that are either in different planes (tilted bipolar) or in the same plane (monopolar focused) while *TPX2* decorates MTs in close proximity to the centrosomes (Figure 1b). We also observed an “unfocusing” of the unique spindle pole, characterized by γ-tubulin diffuse staining in a ring-like structure similar to *TPX2* staining (monopolar unfocused). 

We next measured the mitotic index of si*ATAT1* and siLuc cells (Figure 1c). As expected from the *ATAT1* depletion-induced mitotic phenotype, there were about twice as many mitotic cells in si*ATAT1* cells than in siLuc cells, indicating that si*ATAT1* cells spent more time in mitosis. Direct measurement of mitotic length from nuclear envelope breakdown (NEBD) to anaphase, in live GFP-tubulin-expressing LLCPK1 cells, confirmed that most si*ATAT1* cells took far longer to align chromatids on the metaphase plate and to induce anaphase onset (Figure 1d).

To control for off-target effects of the siRNA, we engineered doxycycline inducible GFP-wild type (wt) and catalytic inactive (dead) *ATAT1*-LLCPK1 cell lines in which the transgenes are resistant to the siRNA. Upon depletion of endogenous *ATAT1*, re-expression of the wt-*ATAT1* resulted in partial rescue of the monopolar spindle phenotype while no significant rescue was observed upon re-expression of the dead form of *ATAT1* (Figure 1e). We do not fully understand why only a partial (but reproducible) rescue of the spindle phenotype is observed following re-expression of wt-*ATAT1*. Potential explanations include that *ATAT1* access to a specific subset of MTs (such as centriolar MTs) may require more time. Although some functions of *ATAT1* are independent of its catalytic activity [13], we show that control of spindle bipolarity requires the catalytic activity of *ATAT1* in LLCPK1 epithelial cells.

### 3.2. Kinesin-5 Activity Is Not Affected in siATAT1 Cells

**Figure 2:**
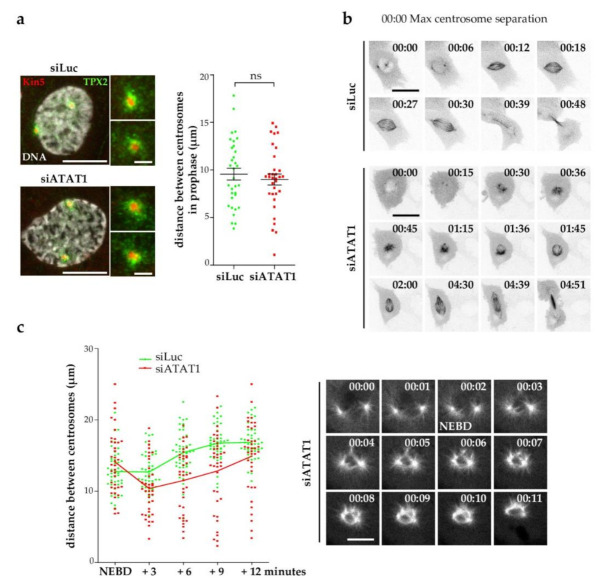
Kinesin-5 dependent centrosome separation is not affected in si*ATAT1*cells. LLCPK1 (**a**) or GFP-tubulin-expressing LLCPK1 cells (**b**,**c**) were transfected for 48 h with siRNAs directed against luciferase (Luc) or *ATAT1*. (**a**) Representative MIP images of siLuc and si*ATAT1* prophase cells stained for kinesin-5, *TPX2*, and DAPI. Scale bar is 10 µm. Insets show detailed areas of both centrosomes (scale bar is 2.5 µm). Distances between the two centrosomes were measured, in both conditions, on 3D stacks using Imaris software. Around 10 prophases were counted per condition and per experiment (*n* = 3). (**b**) Live cells were imaged every 3 min using a dragonfly ANDOR spinning disk confocal microscope. Scale bar is 25 µm. (**c**) Left panel, quantification of the evolution of the distance between the two centrosomes in a 12-min time range after nuclear envelop breakdown (NEBD). Distances were measured as in (**a**) (*n* = 3, in total 49 siLuc, and 37 si*ATAT1* cells were analyzed). Solid lines show the mean of the distances. Right panel, representative images, acquired every min, of an si*ATAT1* GFP-tubulin-expressing LLCPK1 cell entering mitosis. Scale bar is 10 µm.

In prophase, starting roughly one hour before NEBD, the activity of kinesin-5 (KIF11/Eg5) regulates the optimal separation of centrosomes and insures the formation of an efficient bipolar spindle [39]. Centrosomal recruitment and activity of kinesin-5 depends upon *TPX2* and NEK9 activity [40] and its chemical inhibition by STLC or its downregulation results in the formation of monopolar spindles [41,42]. To analyze whether kinesin-5 activity might be hampered by the lack of MT acetylation, we measured the distance between centrosomes of prophase siLuc and si*ATAT1* cells. Centrosome separation before NEBD is not affected by loss of *ATAT1*, a finding confirmed by similar staining of the recruitment of both kinesin-5 and its regulator *TPX2* to centrosomes (Figure 2a).

We next analyzed by live imaging, using GFP-tubulin-expressing LLCPK1 cells, how monopolar spindles formed in si*ATAT1* mitotic cells. In siLuc cells, mitosis completion occurred within one hour after NEBD (Figure 2b, top panel and Video S1). In live si*ATAT1* cells, as for siLuc cells, initial centrosomes separation occurred, but after NEBD, centrosomes come closer to each other. While a number of these spindles coped with the stress and evolved as bipolar spindles (Figure 2b, bottom panel and Video S2), others remained monopolar for several hours. Other si*ATAT1* cells formed bipolar spindles that were very mobile and tilted, and centrosomes sometimes detached from and reattached to the spindle (Video S3).

Since centrosomes appeared to get closer together after NEBD in si*ATAT1* cells, we quantified the distance between centrosomes at shorter time intervals after NEBD in live siLuc and si*ATAT1* cells (Figure 2c). Despite heterogeneity, the distance between the two centrosomes in the siLuc cells remained similar in the first three minutes after NEBD and then increased as the bipolar spindle was established. In contrast in si*ATAT1* cells, a shortening of the distance between the two centrosomes occurred in the first three minutes after NEBD. This is illustrated by GFP-tubulin behavior of an si*ATAT1* cell in which the MT nucleation centers get closer to each other for several minutes after initial separation (Figure 2c).

### 3.3. Centrosome Maturation and Centrosome-Dependent MT Nucleation Are Defective in siATAT1 Mitotic Cells

**Figure 3:**
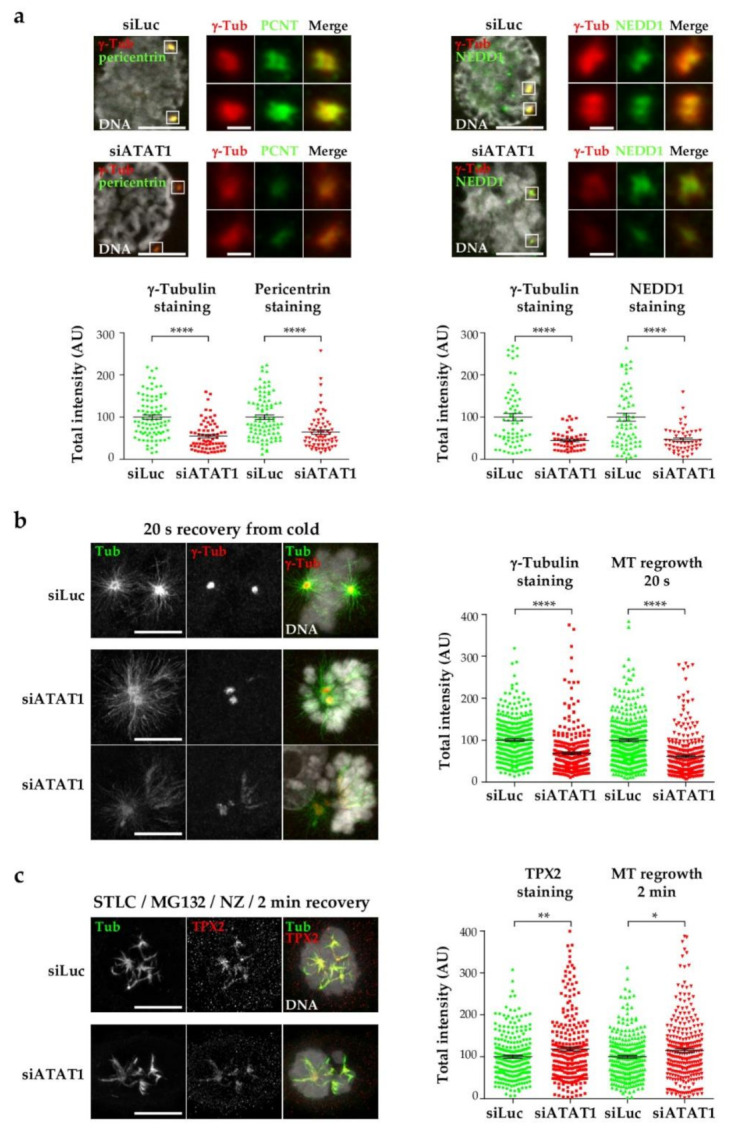
Centrosome maturation and nucleation are deficient in si*ATAT1* cells. LLCPK1 cells were transfected for 48 h with siRNAs directed against luciferase or *ATAT1*. (**a**) Cells were fixed and processed for immunofluorescence. Representative MIP images of prophase siLuc and si*ATAT1* cells stained for γ-tubulin and pericentrin (left panels) and γ-tubulin and NEDD1 (right panel) are shown. Scale bars are 9 µm. Insets show higher magnification of the centrosomes regions (scale bars are 2 µm). Total γ -tubulin, pericentrin, and NEDD1 levels were quantified using image J software. At least 15 cells were analyzed per condition and per experiment (*n* = 3). (**b**) Cells were cold treated for 20 min and then warmed for 20 s before fixation. Left panels: representative MIP images of mitotic siLuc and si*ATAT1* cells stained for tubulin, γ-tubulin, and DNA. Scale bars are 7.5 µm; Right panel: quantification of total γ-tubulin and tubulin intensities around centrosomes after 20 s recovery from cold. At least 100 cells were analyzed per condition and per experiment (*n* = 3). (**c**) Cells were treated for 24 h with 2 µM STLC after 24 h siRNA treatment and then washed and released in MG132 (10 µM) for 2 h. MTs were then depolymerized for 1 h with nocodazole (NZ, 2 µg/mL) and MT regrowth was allowed for 2 min at 37 °C after washing away NZ and cells were fixed. Left panels: representative MIP images of mitotic siLuc and si*ATAT1* cells stained for *TPX2*, tubulin and DNA. Scale bar is 5 µm; Right panel: quantification of total *TPX2* and tubulin intensities around mitotic chromosomes after 2 min recovery from nocodazole. At least 80 cells were analyzed per condition and per experiment (*n* = 3). *p* < 0.0001 is considered highly significant and noted in the graphs with ****. Between 0.001 and 0.01 with ** and between 0.01 and 0.05 with *.

Failure of efficient centrosome separation at mitotic entry could result from poor MT nucleation. Centrosomes are the main MT nucleation centers in somatic cells. After the G2/M transition, recruitment of more pericentriolar material embeds the γ-tubulin ring complexes in a matrix and promotes MT nucleation. We thus analyzed the recruitment to the centrosomes of the main MT nucleation factor γ-tubulin, NEDD1 that binds γ -TURC, and of the pericentriolar matrix protein pericentrin. Interestingly, the recruitment of γ-tubulin, NEDD1 and pericentrin appeared defective in si*ATAT1* prophase centrosomes (Figure 3a), which could indicate that centrosome maturation is affected by lack of MT acetylation. We therefore looked at the MT nucleation activity at mitotic centrosomes. SiLuc and si*ATAT1* cells were cold-treated to depolymerize MTs, and allowed to warm for 20 s to induce ‘de novo’ MT nucleation [43]. Representative images are shown (Figure 3b). Quantification of the signals showed that both γ-tubulin and MT total intensities in the immediate vicinity of the γ-tubulin dots were decreased in si*ATAT1* compared to siLuc cells demonstrating that γ-tubulin recruitment and “de novo” MT nucleation from centrosomes are hampered in the absence of *ATAT1*.

We next analyzed MT nucleation from chromosomes and kinetochores, since, unlike MT nucleation from centrosomes, this depends upon the release of spindle assembly factors by the RCC1-generated gradient of Ran-GTP [43,44]. To measure MT nucleation from chromosomes, we increased the number of mitoses by synchronizing the cells in prometaphase/metaphase. For this, we used the kinesin-5 inhibitor STLC and then released the cells in the presence of the proteasome inhibitor MG132 to allow spindle bipolarization and prevent metaphase to anaphase transition. Cells were then treated with nocodazole to depolymerize the MT network and fixed after two minutes of recovery from nocodazole. Representative images of *TPX2* and tubulin stainings show that MT nucleation from chromatin was in a similar range in siLuc and si*ATAT1* cells. Quantification of the total MT intensity around the chromosomes confirmed that MT nucleation was similar with a slight but significant increase in si*ATAT1* compared to siLuc cells (Figure 3c). This robust MT nucleation around chromatin may explain why a number of si*ATAT1* cells cope with defects and terminate mitosis.

In summary, our data show that the mitotic centrosome maturation and the centrosome-induced MT nucleation capacity are defective in the absence of MT acetylation while chromatin-induced MT nucleation is not lowered.

### 3.4. PLK1 Recruitment to Centrosomes Is Hampered in siATAT1 Cells

**Figure 4:**
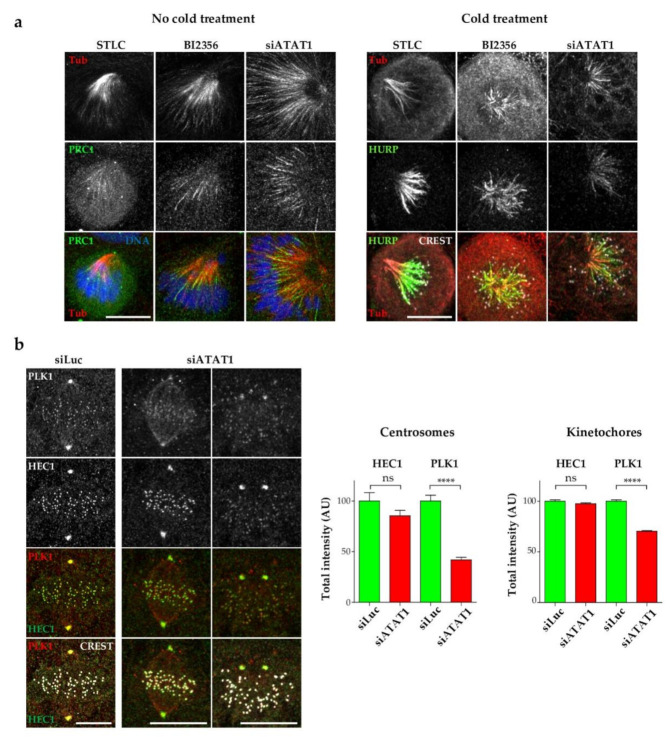
*ATAT1* depletion affects *PLK1* recruitment at centrosomes. (**a**) LLCPK1 cells were transfected for 48 h with siRNAs directed against luciferase or *ATAT1*. SiLuc cells were treated after 24 h with STLC (2 µM) for 24 h or after 46 h with BI2356 (100 nM) for 2 h. Cells were either fixed immediately (left panels) or after 1 h cold treatment (right panels) and processed for immunofluorescence. Left panels: Representative MIP images of monopolar spindles (resulting from STLC, BI2356 or si*ATAT1* treatments) stained for tubulin, PRC1 and DNA. Scale Bar is 10 µm. Right panels: Representative MIP images of the monopolar spindles, after 1 h cold treatment, stained for tubulin, the K-fiber marker HURP and DNA. Scale bar is 10 µm. (**b**) LLCPK1 cells transfected for 48 h with siRNAs directed against luciferase or *ATAT1* were fixed and stained for *PLK1*, *HEC1*, and CREST. Representative MIP images of an siLuc metaphase spindle and si*ATAT1* bipolar and monopolar spindles are shown. Quantification of total *HEC1* and *PLK1* intensities at centrosomes and at every kinetochore was performed on 3D stack images using Imaris software. At least 25 centrosomes and 700 kinetochores were analyzed per condition and per experiment (*n* = 3). *p* < 0.0001 is considered highly significant and noted in the graphs with ****.

The main mitotic kinase *PLK1* is involved in multiple processes throughout mitotic progression [27]. Most importantly, *PLK1* regulates centrosome maturation, MT nucleation at centrosomes, and MT-kinetochore attachments and stability. *PLK1* inhibition, by its highly specific inhibitor BI2356, results in bipolar spindle collapse [45]. We thus tested whether MT acetylation levels may be important for efficient *PLK1* activity. We first compared the characteristics of monopolar spindles induced by inhibition of kinesin-5 (STLC) or *PLK1* (BI2356) or by depletion of *ATAT1*. While MTs in STLC-induced monopolar spindles did not extend beyond the chromosomes, long MTs extended far away from the chromosome plate in BI2356 monopolar spindles, a phenotype similar to that of si*ATAT1* cells (Figure 4a, left panels). In addition, the anti-parallel MT bundler PRC1 strongly stained MTs bundles both in BI2356-treated and si*ATAT1* cells but not in STLC-induced monopolar spindles (Figure 4a, left panels).

Next, BI2356, STLC, and si*ATAT1*-treated cells were subjected to a brief cold treatment and fixed to visualize only the stable MT-kinetochore-fibers (K-fibers). HURP protein accumulation on K-fibers was reported to be inversely proportional to their length by a mechanism that is controlled by centrosomes [46]. In STLC-treated cells, a sharp and restricted region close to the plus end of the K-fibers was decorated by HURP. In contrast, HURP staining in both BI2356-treated and *ATAT1*-depleted cells extended very close to the MT minus end towards the centrosome region (Figure 4a, right panels). Thus, monopolar spindles induced in si*ATAT1* cells display striking similarities with *PLK1*-inhibited monopolar spindles.

Next, to study *PLK1* recruitment in si*ATAT1* mitotic cells, we stained *PLK1* together with *HEC1* and the CREST marker of centromeres. *HEC1* is a kinetochore protein belonging to the NDC80 complex required for end-on kinetochore-MT attachment, and is also bound to centrosomes [47]. In siLuc metaphase cells, as expected, *PLK1* was recruited to both centrosomes and kinetochores and clearly colocalized with *HEC1*. In both monopolar and bipolar spindles induced by *ATAT1* depletion, centrosomal *PLK1* staining appeared to be much weaker than in siLuc cells (Figure 4b, left panel). Kinetochore *PLK1* recruitment appeared less affected. Quantification of the staining intensity of *HEC1* and *PLK1* at centrosomes and at every kinetochore of siLuc and si*ATAT1* mitotic spindles showed that *HEC1* binding to centrosomes and kinetochores appeared similar in mitotic siLuc and *ATAT1* cells. In contrast, *PLK1* levels were strongly reduced at centrosomes in si*ATAT1* cells and to a lesser extent at kinetochores (Figure 4b, right panel). We thus provide evidence that MT acetylation is required for efficient recruitment of mitotic *PLK1* to centrosomes, and to a lesser extent to the kinetochores.

### 3.5. Loss of MT Acetylation Impedes MT Flux in Mitosis and Induces Breaks in Spindle MT Bundles

**Figure 5:**
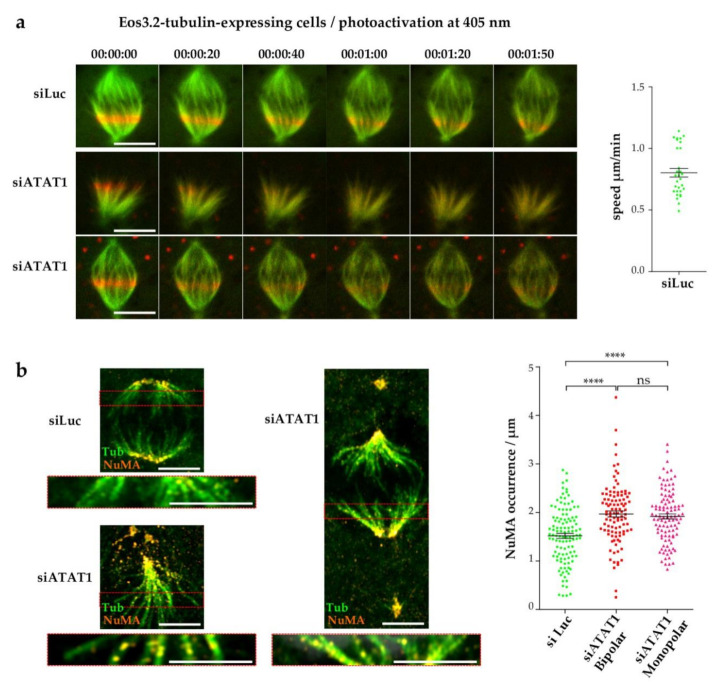
*ATAT1* depletion perturbs the MT spindle flux and induces MT breaks. (**a**) Eos3.2-tubulin-expressing LLCPK1 cells were transfected for 48 h with siRNAs directed against luciferase or *ATAT1*. A defined region, close to the chromosomes of mitotic cells, was photoactivated at 405 nm and images were acquired every 10 s. Sequential and representative images are shown. Scale bar is 9 µm. Flux speed of siLuc cells was estimated by measuring the shortening of the distance from the centrosome to the middle of the red photoactivated tubulin signal, as a function of time (30 cells). Flux speed of si*ATAT1* cells could not be evaluated because of diffuse signal. (**b**) LLCPK1 cells were transfected for 48 h with siRNAs directed against luciferase or *ATAT1*, treated with cold for 20 min, fixed, and immunostained for NuMA and tubulin. Left panels: Representative MIP images of bipolar siLuc and si*ATAT1* spindles and of an si*ATAT1* monopolar spindle are shown. Scale bar is 5 µm. A single plane of the red insets is enlarged below the MIP images. Scale bar is 4 µm. To better visualize NuMA dots along the kinetochore fibers, NUMA staining is represented using the ‘Orange hot’ LUT of Fiji. Right panel: Quantification of NuMA dots along K-fibers. The amount of NuMA dots per individual MT bundle was counted from different cells (>30 MT bundles per condition and per experiment, *n* = 3). *p* < 0.0001 is considered highly significant and noted in the graphs with ****.

In mitosis, the MT flux regulates spindle length. Earlier models suggested that KIF2A-dependent depolymerization of MTs at their minus ends and CLASP1/2-induced MT polymerization at their plus ends is most important for efficient flux [48,49]. More recently, four distinct kinesins were proposed to regulate MT flux, in response to MCAK-mediated depolymerization of K-fibers [50]. Since KIF2A and MCAK are regulated by *PLK1* kinase [51,52,53,54], we analyzed how *ATAT1* depletion affects MT flux. To do so, we used LLCPK1 cells expressing Eos3.2-tubulin that can be photoactivated [35]. Images were recorded every 10 s as the position of the red photoactivated tubulin moved with the flux. MT flux was readily seen in siLuc cells, and the average speed was calculated to be around 0.8 µm/min, in agreement with published reports [55] (Figure 5a top panels, quantification on the right). In contrast, for the bipolar and monopolar spindles analyzed in si*ATAT1* cells, flux was hardly observed. Instead, the photoactivated tubulin appeared to rapidly diffuse throughout the spindle, as if αβ-tubulin heterodimers were reincorporated along the spindle MT lattice (Figure 5a, bottom panels). This suggests that breaks occurred along the spindle MT fibers in si*ATAT1* cells. This finding is consistent with the fact that acetylation of MTs increases their flexibility, bending capacities, and resistance to mechanical breakage [11].

NuMA recruitment to MT minus ends of newly damaged MTs in K-fibers allows, in concert with IFT88, their reanchoring into the spindle [56]. Thus, NuMA dots along K-fibers can be used as a marker of K-fiber integrity. We used this approach and manually counted the number of NuMA dots along K-fibers, outside the centrosome pole region. Staining of siLuc and si*ATAT1* bipolar spindles and an si*ATAT1* monopolar spindle with NuMA and tubulin antibodies (Figure 5b) showed more numerous NuMA dots in si*ATAT1* mitotic cells (insets, Figure 5b) which was confirmed by quantification (Figure 5b, right panel). Thus, NuMA decoration of K-fibers increases in both monopolar and bipolar si*ATAT1* spindles, showing that K-fibers are damaged in si*ATAT1* cells.

### 3.6. Kinetochore Fibers Are Acetylated and Loss of MT Acetylation Activates the Spindle Checkpoint

**Figure 6:**
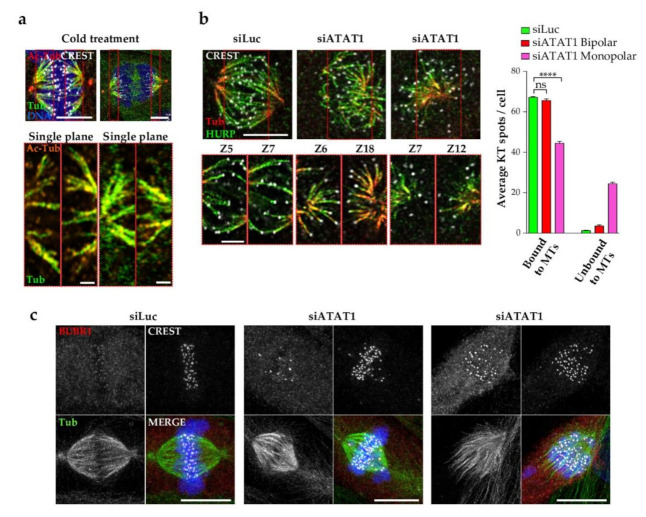
Loss of K-fiber acetylation affects MT-Kinetochore attachments and activates the spindle checkpoint. (**a**) LLCPK1 cells treated with cold for 20 min, and were fixed and immunostained for acetyl-tubulin, tubulin, the centromere marker CREST, and DNA. Representative MIP images of a metaphase and anaphase cells are shown. Scale bars are 5 µm. Single planes of red insets displaying merged staining of acetyl tubulin (orange hot LUT) and tubulin (green) are enlarged below the MIP images. Scale bars are 2.5 µm. (**b**,**c**) LLCPK1 cells transfected for 48 h with siRNAs directed against lucriferase and *ATAT1*. (**b**) Cells were treated with cold for 20 min, fixed and immunostained for tubulin, CREST, and the K-fiber marker HURP. Representative MIP images (scale bar is 5 µm) and enlarged insets of single planes (scale bar is 2.5 µm) of bipolar siLuc and si*ATAT1* spindles and of an si*ATAT1* monopolar spindle are shown. Attachment of kinetochores to MTs was quantified per cell on 3D images using Imaris software. Over 15 spindles per condition and per experiment (*n* = 3) were analyzed. (**c**) Representative MIP images of cells immunostained for DNA, tubulin, CREST, and the spindle assembly checkpoint marker BUBR1 are shown. Scale bars are 10 µm. *p* < 0.0001 is considered highly significant and noted in the graphs with ****.

K-fiber stability and interaction with kinetochores (KTs) are regulated by *PLK1* and a number of its substrates. Since K-fiber integrity appears to be affected in the absence of MT acetylation, we wondered whether tubulin acetylation occurs on K-fibers. Indeed, immunofluorescence staining of cold-treated LLCPK1 cells confirms that tubulin acetylation decorates K-fibers of both metaphase and anaphase mitotic spindles, as seen on MIP images and single planes (Figure 6a). However, MT acetylation is not required for K-fiber formation as HURP staining is observed in si*ATAT1* mitotic cells (Figure 4a).

To address whether MT acetylation is required for efficient KT binding to K-fibers, siLuc and si*ATAT1* cells were depolymerized by cold treatment and K-fibers were stained for tubulin, CREST as a marker for KTs and HURP as a marker of K-fibers. Representative images show that K-fibers are formed in both monopolar and bipolar si*ATAT1* spindles (Figure 6b). Single plane images show better details of the interaction of HURP-bound K-fibers to KTs. We quantified the number of KTs bound/unbound to K-fibers per cell and showed that most KTs were bound to K-fibers in siLuc cells. Interestingly, no strong attachment defect was observed in si*ATAT1* bipolar spindles, although, the quality of the attachment cannot be assessed. However, in si*ATAT1* monopolar spindles, strong defects in attachment were observed. Thus, MT acetylation is required for efficient KT-MT attachment (Figure 6b).

Finally, inefficient KT-MT attachments in si*ATAT1* mitotic cells were confirmed by staining with the spindle checkpoint marker BUBR1 together with tubulin (Figure 6c).

### 3.7. In siATAT1 Cells, Stabilizing MT Plus-Ends, or Increasing Centrosomal PLK1 Activity Restore Spindle Bipolarity

Our results show that MT acetylation in mitosis is important for centrosomal *PLK1* recruitment and centrosome-mediated MT nucleation as well as for K-fiber integrity. To try to discriminate the functions of MT acetylation in the regulation of mechanical properties of MTs from functions linked to *PLK1* defective centrosomal recruitment, we developed several approaches.

**Figure 7:**
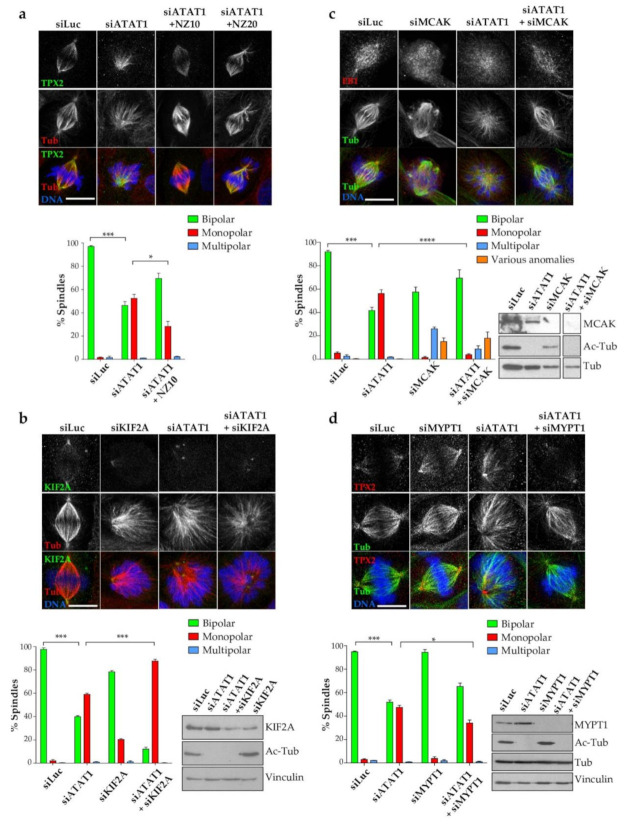
MT-plus-end stabilization and restoring centrosomal *PLK1* activity rescue the si*ATAT1*-induced monopolar spindle phenotype. (**a**) LLCPK1 cells were transfected for 48 h with siRNAs directed against luciferase and *ATAT1*. *ATAT1*-depleted cells were further treated with nothing or with low doses of nocodazole (10 or 20 ng/mL) for 5 h before fixation and immunostaining for tubulin, *TPX2*, and DNA. Representative MIP images are shown (scale bar 12.5 µm). Sorting of the spindle phenotypes in three classes (bipolar, monopolar, multipolar) was done by blind manual counting (>200 mitotic cells per condition and per experiment, *n* = 3). (**b**) LLCPK1 cells were transfected for 48 h with siRNAs directed against luciferase, *ATAT1*, KIF2A, or a combination of KIF2A and *ATAT1*. Cells were either lysed or fixed. Cell lysates were analyzed by western blot using acetyl-tubulin and KIF2A antibodies to estimate depletion efficiencies and anti-vinculin as loading control. Fixed cells were immunostained for tubulin, KIF2A, and DNA. Representative MIP images are shown (scale bar 8 µm). Phenotype quantification was performed as in (**a**). (**c**) LLCPK1 cells were transfected for 48 h with siRNAs directed against luciferase, *ATAT1*, MCAK, or a combination of MCAK and *ATAT1*. Cells were either lysed or fixed. Cell lysates were analyzed by western blot using acetyl-tubulin and MCAK antibodies to estimate depletion efficiency and anti-tubulin as loading control. Fixed cells were immunostained for the MT plus tip marker EB1, tubulin and DNA. Representative MIP images are shown (scale bar 12.5 µm). Sorting of the spindle phenotypes in four classes (bipolar, monopolar, multipolar, various anomalies) was done by blind manual counting (>200 mitotic cells per condition and per experiment, *n* = 3). (**d**) LLCPK1 cells were transfected for 48 h with siRNAs directed against luciferase, *ATAT1*, the PP1C targeting subunit MYPT1 or a combination of MYPT1 and *ATAT1*. Cells were either lysed or fixed. Cell lysates were analyzed by western blot using acetyl-tubulin and MYPT1 antibodies to insure depletion efficiency and anti-vinculin and anti-tubulin antibodies as loading controls. Fixed cells were immunostained for *TPX2*, tubulin, and DNA. Representative MIP images are shown (scale bar 10 µm). Phenotype quantification was performed as in (**a**). *p* < 0.0001 is considered highly significant and noted in the graphs with ****. P Values between 0.0001 and 0.001 are annotated with ***; And between 0.01 and 0.05 with *.

First, we asked whether regulation of overall spindle MT dynamics could rescue the monopolar spindle phenotype of si*ATAT1* cells. To do so, we treated cells with low doses of nocodazole (10–20 ng/mL) which reduces MT dynamics [48]. Representative images of spindles stained for *TPX2* and tubulin are shown (Figure 7a). Quantification shows that lowering the dynamics of spindle MTs in si*ATAT1* cells with 10 ng/mL nocodazole resulted in a significant rescue of the monopolar spindle phenotype (Figure 7a). Although slightly higher doses of nocodazole also restored spindle bipolarity, it impaired chromosome congression to the metaphase plate, indicating that MT dynamics must be tightly controlled (Figure 7a, NZ20).

Second, we explored the fact that loss of the centrosomal KIF2a depolymerase induces monopolar spindles. As in si*ATAT1* cells, the KIF2A-knockdown-induced monopolar phenotype was rescued by reducing MT dynamics with nocodazole [48]. We therefore wondered whether stabilization of spindle MT minus-ends could affect the si*ATAT1*-induced mitotic phenotype. SiLuc, si*ATAT1*, siKIF2A, and si*ATAT1*+siKIF2A cells were analyzed by western blot (Figure 7b). As expected, in si*ATAT1* and si*ATAT1*+siKIF2A cells acetylated MTs were no longer detected. By comparison to siLuc and si*ATAT1* cells, the level of KIF2A was only reduced by a factor of two in siKIF2A and si*ATAT1*+siKIF2A cells showing that the efficiency of KIF2a siRNA was not optimal. Immunofluorescence staining and quantification showed that around 20% of the mitotic spindles induced in siKIF2A cells were monopolar and had reduced KIF2A staining at the poles. In si*ATAT1* cells, around 60% of the spindles were monopolar and depending on the focusing of the poles KIF2A staining could be resolved as two dots or was more diffuse. Surprisingly, in si*ATAT1*+siKIF2A co-depleted cells, mitotic monopolar spindle formation sharply increased to over 80% of all spindles (Figure 7b). Thus, stabilization of MT spindle minus-ends does not rescue the si*ATAT1*-induced monopolar spindles. On the contrary, additive effects of the double depletion on spindle monopolarization indicate that *ATAT1* and KIF2A regulatory pathways are distinct.

Next, since centrosome integrity and KT-MT interactions are affected by *ATAT1* loss, we wondered whether stabilizing KT attachment to MTs by depleting the KT-bound MT depolymerase MCAK could rescue the monopolar spindles of si*ATAT1* cells. MCAK depolymerase activity is regulated by *PLK1* [54] and its depletion induces lagging chromosomes, longer spindles and various anomalies [57]. In siMCAK and siMCAK+si*ATAT1* cells, MCAK was no longer detected by western blot and acetylated tubulin was undetectable in si*ATAT1* and siMCAK+si*ATAT1* cells (Figure 7c). Representative images of depleted cells stained for total tubulin and the MT plus-end marker EB1 are shown (Figure 7c). Spindle phenotype quantification shows that approximately 40% of the siMCAK-spindles are abnormal. Among these, over 20% are bipolar with small extra poles (classified here as multipolar). No monopolar spindles were induced by MCAK depletion. In si*ATAT1* cells, as expected, almost 60% of the mitotic spindles were monopolar. Co-depletion of both proteins restored spindle bipolarity in almost 70% of the cells. These results show that loss of spindle MT acetylation is compensated for when defective MT-KT attachments, observed in si*ATAT1* cells (Figure 7c), are stabilized by co-depleting MCAK allowing spindle bipolarization.

Finally, we wondered whether the observed restoration of spindle bipolarity by stabilization of MT-KT attachments merely reflects the higher sensitivity of deacetylated MTs to breakage or may also depend upon the defective centrosomal *PLK1* observed in si*ATAT1* cells. Indeed, *PLK1* regulates KT-MT attachment and centrosomes were shown to regulate K-fiber dynamics [46]. To answer this question, we manipulated the targeting of the phosphatase PP1c which antagonizes the centrosomal activity of *PLK1*. Indeed, the phosphatase regulatory subunit MYPT1 targets PP1c to centrosomes and its depletion was shown to increase *PLK1* phosphorylation at its activating site and to rescue the monopolar spindle phenotype induced by *PLK1*-inhibition [31]. To analyze whether monopolar spindles induced in si*ATAT1* cells resulted from the decreased centrosomal *PLK1* accumulation observed in these cells, we reinstated *PLK1* activity in si*ATAT1* cells by codepletion with MYPT1. Western blot analyses confirmed that MYPT1 was efficiently depleted in siMYPT1 and siMYPT1+si*ATAT1* cells, and that acetyl-tubulin levels remained undetectable in both si*ATAT1* and siMYPT1+si*ATAT1* cells (Figure 7d). MYPT1 depletion alone did not induce strong spindle morphology defects, while approximately 50% of the mitotic spindles were monopolar in si*ATAT1* cells. Co-depletion of MYPT1 together with *ATAT1* rescued the monopolar spindle phenotype, showing that increasing *PLK1* activity by depleting MYPT1 [31] is sufficient to oppose the loss of microtubule acetylation in mitotic cells.

## 4. Discussion

Different MT post-translational modifications decorate subsets of the MT mitotic spindle. MT polyglutamylation and detyrosination on the C-terminal tubulin tails is directly involved in binding MAPs and motors. While the function of polyglutamylation during mitosis has not been reported, highly specific functions of MT detyrosination have been documented. This MT modification controls spindle orientation by regulating cortical anchoring via the Cap-Gly domain-containing plus tips, CLIP170 and p150-Glued [6], chromosome congression by guiding the kinesin CENP-E to the right MT tracks [7], and error correction by controlling centromeric MCAK activity [8]. MT acetylation occurs in the lumen of the MT and is thus unlikely to directly control the binding of MAPs and motors. It has been shown to increase MT flexibility and thus resistance to mechanical breakage [10] and to regulate MT architecture [58]. However, no mitotic functions were, as yet, directly attributed to this modification. Nevertheless, to achieve spindle organization, MTs are submitted to extensive tracking and pushing forces as well as torques [59] and thus MT acetylation may be required to resist these mechanical constrains. We thus decided to analyze whether MT acetylation of subsets of spindle MTs contributes to mitotic spindle plasticity.

Using an epithelial cell model, we found that depletion of the MT acetyltransferase *ATAT1* prevents MT acetylation and results in severe mitotic defects, chiefly characterized by the formation of monopolar spindles.

We found that mitotic K-fibers are acetylated and that their integrity is compromised in *ATAT1*-depleted cells. Indeed, in the absence of MT acetylation, the K-fibers are highly decorated with NuMA, a protein recruited to broken K-fibers in order to promote their reincorporation into the spindle [56]. Breaks also likely occur in the bundles on interpolar spindle MTs, as photoactivated tubulins in the spindle MTs of si*ATAT1* cells do not flux but rather diffuse throughout the spindle. In addition, stabilization of spindle MTs with low doses of nocodazole, rescues the mitotic spindle bipolarization. Together, these results provide evidence for a role for MT acetylation in regulating the plasticity of the mitotic spindle. Nevertheless, it is difficult to explain the monopolar spindle phenotype observed in si*ATAT1* cells solely by this mechanical function.

Different mechanisms regulate spindle bipolarization. CDK1-dependent activation of kinesin-5 is the main trigger of centrosome separation in late G2 [60], although additional pathways exist [61,62]. Inactivation of kinesin-5 does not prevent mitotic entry but leads to the establishment of monopolar mitotic spindles with two nearby spindle poles. However, in si*ATAT1* cells, centrosome separation is not affected until NEBD, ruling out major defects in kinesin-5 activity. Instead, we observed that in many si*ATAT1* cells, in the few minutes after NEBD, the two centrosomes come closer together and form a monopolar spindle which may remain as such for hours or evolve to generate bipolar spindles.

NEBD releases the chromosomes and allows MTs to bind to their kinetochore region. While K-fibers still form in the absence of MT acetylation, their attachment to kinetochores is defective and the spindle checkpoint is activated. Interestingly, spindle bipolarization can be restored in si*ATAT1* cells by stabilizing the MT plus-ends of K-fibers by co-depleting MCAK depolymerase, while stabilizing their minus ends by co-depleting the centrosomal KIF2A depolymerase increased monopolarity. It has been recently shown that perturbation of MT-KT interaction following the removal of CENP-A induces loss of mitotic spindle integrity and PCM dispersion [63]. We found that MT acetylation regulates centrosome maturation as accumulation of pericentrin, which acts as a scaffold for recruitment of other components of the pericentriolar material [64,65], is hampered in si*ATAT1* cells. Recruitment and docking of the γ-tubulin ring complex depends upon NEDD1, CDK5RAP2, and *PLK1* [66,67,68,69,70]. We demonstrate that NEDD1, *PLK1*, and γ-tubulin recruitment to centrosomes are disrupted by the absence of MT acetylation, a finding confirmed by the decreased capacity of si*ATAT1* mitotic centrosomes to renucleate MTs after a cold treatment. The initiation of centrosome maturation requires *PLK1*-mediated phosphorylation of pericentrin [71] and loss of either pericentrin or *PLK1* reduces MT nucleation at mitotic onset and induces monopolar spindles [45,72]. The formation of monopolar spindles in si*ATAT1* cells thus likely results from loss of centrosome integrity. One possible cause of loss of centrosome integrity is that lack of MT acetylation on the K-fibers prevents correct MT-KT attachments which in turn affects centrosome maturation. Other possibilities are that loss of acetylation of the centriole MTs is sufficient to impair centrosome maturation or that the acetylated spindle MTs serve as tracks to correctly localize centrosomal components. A recent study in Drosophila shows that deletion of *ATAT1* affects centriole size supporting these hypotheses [73]. Defects in centrosome maturation could in turn affect MT-KT attachments. Indeed, centrosomes have been shown to regulate K-fiber plus-end dynamics and length by controlling the level of HURP recruited to the fibers [46]. Moreover, higher MT acetylation levels have been found associated with the older centrosome and the presence of cenexin specifically on the older centrosome has been linked to an increased KT-MT stability [74].

We found that *PLK1* accumulation at centrosomes and to a lesser extent at kinetochores is impaired in the absence of MT acetylation. *PLK1* has numerous substrates at both subcellular locations to ensure proper mitotic progression. Targeting of *PLK1* to centrosomes and kinetochores occurs by different mechanisms. Expressing the polo-box domain (PBD) of *PLK1* is sufficient for efficient targeting to the centrosomes [75] and positive feedback loops exist between centrosomal *PLK1* activation and some of its PBD-binding substrates, such as pericentrin [71]. In contrast, the PBD is not sufficient for *PLK1* targeting to kinetochores which requires additional pathways involving BUB1 and CENP-O [76]. The regulation of *PLK1* activity is also dependent on its localization. At centrosomes, *PLK1* activity is counteracted by the phosphatase PP1C. We show that the monopolar spindle phenotype in si*ATAT1* cells is rescued by solely increasing *PLK1* activity at the centrosomes, using codepletion with MYPT1 a regulatory subunit of PP1C. This means that centrosomal defects may induce the loss of efficient MT capture by kinetochores. Direct phosphorylation of MCAK by centromeric *PLK1* regulates its depolymerase activity at MT plus tips and thereby regulates correct KT-MT attachment and chromosome biorientation [53,77,78,79]. In addition, *PLK1*-dependent phosphorylation of MCAK regulates K-fiber length [78], as we also observed by HURP staining of K-fibers in *PLK1* inhibited and si*ATAT1* depleted monopolar spindles. Thus, both the centrosomal and the centromeric *PLK1* pools that are affected by lack of *ATAT1* can contribute to establishment of incorrect KT-MT interactions.

Several acetyltransferases such as TIP60 or P300/CBP-associated factor have been shown to be important for correct mitotic progression by acetylating various proteins including the MT-plus end binding protein EB1, the kinetochore-associated protein *HEC1*, and the spindle assembly checkpoint protein BubR1 [80,81,82]. Here we demonstrate that cells depleted of the MT-acetyltransferase *ATAT1* display K-fiber breaks, centrosomal maturation defects, and abnormal MT-KT interactions. These defects result in the formation of monopolar spindles. Whether the defects initially result from the lack of resistance of the K-fibers or from defective localization of centrosomal components along the spindle MT tracks still remains an open question. Nevertheless, it is clear that MT acetylation regulates mitotic spindle plasticity and mechanical properties, as well as the dynamic instability of the bipolar MT spindle, by controlling the correct recruitment of mitotic *PLK1* kinase to the centrosomes.

## Data Availability

All plasmids and cell lines described in this study are freely available from Nathalie Morin or Juliette van Dijk.

## Supplementary Materials

The following are available online at https://www.mdpi.com/article/10.3390/cells10081859/s1; Video S1. Live imaging of GFP-tubulin-expressing LLCPK1 cells treated with siLuc. Note that the cell divides within one hour. Frame is 50 µm; Video S2. Live imaging of GFP-tubulin-expressing LLCPK1 cells treated with si*ATAT1* forming a monopolar spindle that evolves to a bipolar spindle and finally divides. Note that the centrosomes come closer to each other after NEBD forming a monopolar spindle. After ~2 h a bipolar spindle is formed and after ~4.5 h cell proceeds with anaphase and terminates mitosis. Frame is 50 µm; Video S3. Live imaging of GFP-tubulin-expressing LLCPK1 cells treated with si*ATAT1* showing a mobile bipolar spindle and centrosome detachment/reattachment. Microtubules form a bipolar spindle that turns and tilts in the cell. After ~5 h, the centrosomes detach. One centrosome reattaches to the opposite spindle pole. After 10 h, the cell had not divided. Frame is 20 µm.
